# Effects of different anticoagulant drugs on the prevention of complications in patients after arthroplasty: A network meta-analysis

**DOI:** 10.1097/MD.0000000000008059

**Published:** 2017-10-27

**Authors:** Ji-Hai Gao, Xiu-Cheng Chu, Lin-Liang Wang, Bo Ning, Chuan-Xin Zhao

**Affiliations:** Department of Joint Surgery, People's Hospital of Dongying, Dongying P.R. China.

**Keywords:** arthroplasty, asymptomatic deep venous thrombosis, CRNM bleeding, prevention, randomized controlled trials

## Abstract

**Background:**

After arthroplasty treatment, some complications commonly occur, such as early revision, infection/dislocation, and venous thromboembolism (VTE). This study aims to use a network meta-analysis to compare effects of 9 anticoagulant drugs (edoxaban, dabigatan, apixaban, rivaroxaban, warfarin, heparin, bemiparin, ximelagatran, and enoxaparin) in preventing postoperative complications in arthroplasty patients.

**Methods:**

After retrieving PubMed, Embase, and Cochrane Library database from the inception to November 2016, randomized controlled trials were enrolled. The integration of direct and indirect evidences was performed to calculate odd ratios and the surface under the cumulative ranking curves. Nineteen eligible randomized controlled trials were included.

**Results:**

The network meta-analysis results showed that compared with warfarin, edoxaban, apixaban, and rivaroxaban had a lower incidence rate in asymptomatic deep venous thrombosis, which indicated that edoxaban, apixaban, and rivaroxaban had better effects on prevention. Similarly, in comparison to enoxaparin, edoxaban and rivaroxaban had better effect; rivaroxaban was better than ximelagatran in preventive effects. Compared with apixaban, edoxaban, dabigatan, rivaroxaban, and enoxaparin had a higher incidence rate in clinically relevant non-major bleeding, which showed that preventive effects were relatively poor. In addition, the results of the surface under the cumulative ranking curves showed that rivaroxaban and bemiparin worked best on symptomatic deep venous thrombosis and pulmonary embolism. In terms of bleeding, apixaban and warfarin had better preventive effects.

**Conclusion:**

Our findings suggested that rivaroxaban may work better in terms of symptomatic deep venous thrombosis and pulmonary embolism, whereas apixaban had better preventive effects in bleeding.

## Abstract 2

Supplemental Digital Content is available in the text

## 1 Introduction

Arthroplasty is 1 commonly used technique for the orthopedic surgical treatment that is refractory to conservative treatment; besides, as the size of the elderly population is increasing, the number of procedures for arthroplasty is also increasing.^[1,2]^ The treatment outcomes after arthroplasty are generally excellent to relieve pain and restore function to the joint, with low complication rates.^[3,4]^ However, some complications of patients who received arthroplasty treatment have significant consequences, such as early revision, infection/dislocation, venous thromboembolism (VTE), and death in comparison with age-matched controls.^[5]^ Lateral release had no effect on patellar subluxation, dislocation, or loosening, but was associated with significantly more patellar fractures.^[6]^ Number of patients combined internal rotation correlated with lateral tracking and patellar tilting, patellar subluxation, and early patellar dislocation or late patellar prosthesis failure.^[7]^ However, a high frequency of asymptomatic pulmonary embolism (PE) in patients with deep venous thrombosis (DVT) has been reported, but information about the outcome of the patients with PE remains sparse.^[8]^ Among patients suspected to have PE, a substantial number had DVT in the absence of PE.^[9]^ Furthermore, safety outcomes included major bleeding, clinically relevant non-major (CRNM) bleeding, and any clinically relevant bleeding (major bleeding plus CRNM).^[10]^

Deep venous thrombosis in many cases led to chronic symptoms in the damaged leg, even though the affected veins had recanalized.^[11]^ In patients with idiopathic DVT, continuing anticoagulant therapy beyond 3 months was associated with a reduced incidence of recurrent thrombosis during the period of therapy.^[12]^ Patients with PE during follow-up underwent transthoracic echocardiography, and, if supportive findings were present, ventilation-perfusion lung scanning and pulmonary angiography.^[13]^ Clinically relevant bleeding (CRB), comprising major bleeding and CRNM, had been used as a surrogate for major bleeding in most anticoagulant trials, but its validity for estimating compromise between thrombotic and bleeding events had never been assessed.^[14]^ Oral anticoagulant therapy was associated with an increased risk of hemorrhage, which could be assessed by bleeding risk scores.^[15]^ Recently, new agent oral anticoagulants have been introduced which make the long-term VTE prophylaxis more comfortable.^[16]^ There are several oral agents that have been studied for the prevention of VET showing significant curative in preventing VET. There were multiple previous randomized trials comparing different oral drugs for the prevention of VTE after arthroplasty. For example, Eriksson et al^[17]^ compared the oral dabigatran etexilate and subcutaneous enoxaparin, indicating that dabigatran etexilate at 220 mg or 150 mg might be effective and safe as enoxaparin for prevention of VTE in those patients after arthroplasty. Furthermore, Lassen et al^[18]^ found that a daily dose of 2.5 mg of apixaban might be more convenient and effective than 40 mg per day enoxaparin management. Moreover, Fuji et al^[19]^ reported the latest findings of their research, which, on the contrary, proved that daily oral administration of edoxaban was superior to subcutaneous enoxaparin in preventing VTE after arthroplasty. However, there was a lack of systematic comparison for the efficacies of multiple types of different oral anticoagulants on complications in patients after arthroplasty. The network meta-analysis is a commonly used comprehensive research method, which can directly summarize the results of pair-wise meta and indirectly do comparative calculation so that different experimental results can be compared with each other and result in a more complete and comprehensive conclusion.^[20]^ Therefore, we intend to perform a network meta-analysis to compare the 9 anticoagulant agents’ preventive effects on preventing postoperative complications in arthroplasty patients.

## 2 Materials and methods

This study was performed in accordance with the Preferred Reporting Items for Systematic Reviews and Meta-Analyses (PRISMA) statement. As it was based on previous publications, it did not require ethical approval or patient consent.

### 2.1 Literature search

We searched electronic scientific literature databases like PubMed, Embase, and Cochrane Library (last updated search in November 2016) to identify studies relevant to the 9 anticoagulant drugs for the prevention of postsurgery complications in joint replacement patients. A combination of keywords and free words were used to retrieve studies relevant to the topic of interest, including lower-extremity DVT (LEDVT), deep venous thrombosis (DVT), arthroplasty, and randomized controlled trials (RCTs).

### 2.2 Eligible criteria

Inclusion criteria were: (1) study design: RCTs; (2) treatments: edoxaban, dabigatan, apixaban, rivaroxaban, warfarin, heparin, bemiparin, ximelagatran, and enoxaparin; (3) study subject: joint replacement patients aged ≥18 years; (4) studies relevant to the prevention of postsurgery complication in joint replacement patients. Exclusion criteria were: (1) patients with bleeding risks in the first 3 months; (2) patients with thrombosis risks (symptomatic DVT or PE, coagulation disorders, and fractures of the lower limbs in the first 6 month, etc); (3) patients with severe renal impairment; (4) patients with liver dysfunction; (5) patients treated with nonartificial joint replacement; (6) literature lack of data integrity (nonpaired study); (7) non-RCTs; (8) repeated publications; (9) meeting reports, system evaluation, or summary articles; (10) non-English documents.

### 2.3 Data extraction and quality assessment

Two investigators independently employed a standardized abstraction form to extract data from eligible trials, and any disagreements were resolved by re-examination of all items and reaching a consensus among several investigators. RCT assessment was conducted by 2 or more of the researchers based on the Cochrane risk-assessment tool,^[21]^ which includes 6 domains: random allocation, allocation concealment, blind method, loss of outcome data, selection of outcome reporting, and other bias. The assessment includes assigning a judgment of “yes,” “no,” or “unclear” for each domain to designate a low, high, or unclear risk of bias, respectively.^[22]^ Publication bias assessment was performed with the use of Review Manager 5 (RevMan 5.2.3, Cochrane Collaboration, Oxford, UK).

### 2.4 Statistical analysis

Traditional pair-wise meta-analyses were first conducted for studies that directly compared different treatment arms. The results were reported as odds ratios (ORs) with 95% confidence interval (95% CI) accounting for study sample sizes. Secondly, R software was used to draw the network diagram among various kinds of intervention measures and different researches, of which each node represents a variety of interventions, the node size of the sample size, and the line weight of nodes of the numbers of included studies. Bayesian network meta-analyses were then performed to compare different interventions with each other. Noninformative priors were enrolled for effect sizes and precision. Convergence and lack of auto correlation were checked and confirmed after 4 chains and a 20,000-simulation burn-in phase; finally, direct probability statements were derived from an additional 50,000-simulation phase.^[23]^ To assist in the interpretation of ORs, we calculated the probability of each intervention being the most effective or safest treatment method based on a Bayesian approach using probability values summarized as surface under the cumulative ranking curve (SUCRA); the larger the SUCRA value, the better the rank of the intervention.^[24,25]^ All computations were done using R (V.3.1.2) package gemtc (V.0.6), along with the Markov Chain Monte Carlo engine Open BUGS (V.3.4.0).

## 3 Results

### 3.1 Baseline characteristics of included study

**Figure 1:**
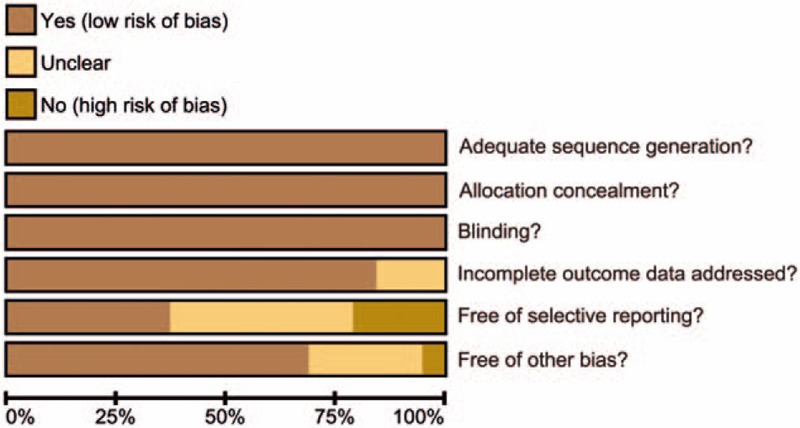
Cochrane system bias evaluation of all the nineteen enrolled studies in this network meta-analysis.

**Figure 2:**
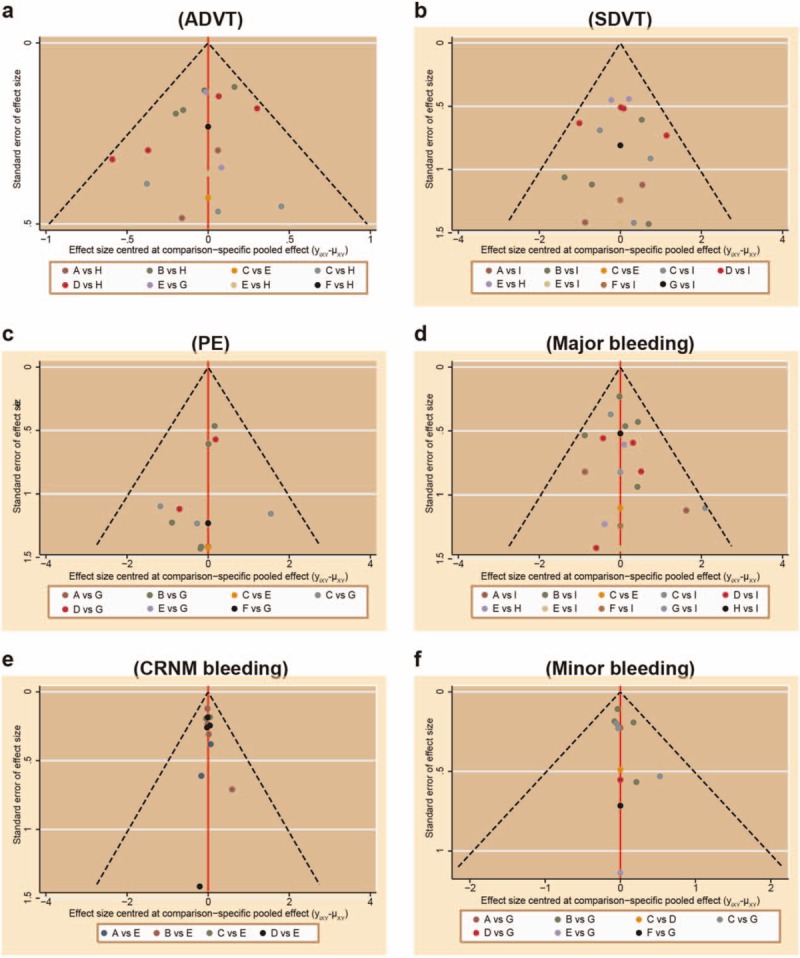
Funnel plot for publication bias of included studies. (A) Edoxaban; (B) dabigatan; (C) apixaban; (D) rivaroxaban; (E) warfarin; (F) heparin; (G) bemiparin; (H) ximelagatran; (I) enoxaparin. ADVT = asymptomatic deep venous thrombosis, CRNM = clinically relevant non-major, PE = pulmonary embolism, SDVT = symptomatic deep venous thrombosis.

Our electronic literature search broadly identified a total of 6732 studies. After reading the titles and abstracts, we excluded 2526 for duplicates, 1048 for letters and reviews, 1001 for non-human studies, 1515 for studies without relation to artificial joint replacement, 538 for studies showing no apparent significance in preventing postsurgery complication in joint replacement patients, 83 for no relation to drug treatment, and 2 for no or incomplete data documents. A total of 19 studies,^[16–19,26–40]^ published between 2002 and 2015, finally met our predetermined inclusion criteria, and were incorporated into our network meta-analysis (Appendix Fig. 1). The baseline characteristics of included studies are displayed in Appendix Table 1. Cochrane system bias evaluation is shown in Fig. 1, which showed a better quality of included RCTs and lower risk of publication bias. Furthermore, the publication bias of included studies was assessed again and generated a funnel plot, which is showed in Fig. 2. It displayed that scatter points distributed in funnel symmetrically, which revealed that there was no significant publication bias.

### 3.2 Pair-wise meta-analysis

**Figure 3:**
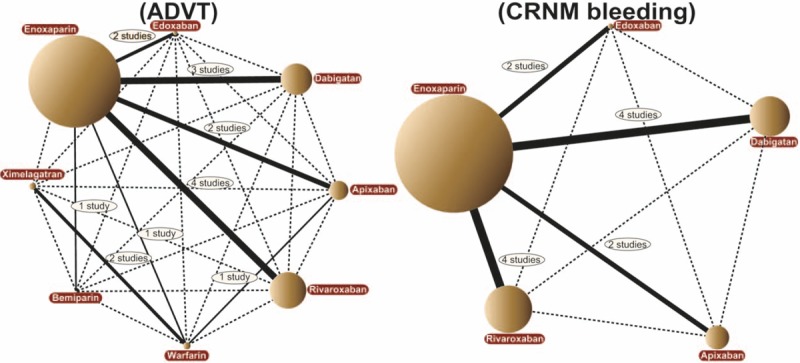
The evidence network of all enrolled studies about the preventive effect of the 9 anticoagulant drugs (edoxaban, dabigatan, apixaban, rivaroxaban, warfarin, heparin, bemiparin, ximelagatran, and enoxaparin) on the ADVT and CRNM bleeding in postoperative patients receiving arthroplasty in this network meta-analysis. ADVT = asymptomatic deep venous thrombosis, CRNM = clinically relevant non-major.

Direct paired comparison showed that patients taking edoxaban, apixaban, and rivaroxaban had a low incidence of ADVT when compared with enoxaparin (OR 0.38, 95% CI 0.23–0.63; OR 0.34, 95% CI 0.16–0.74; OR 0.41, 95% CI 0.22–0.76, respectively), which showed that the preventive effect was better. Also, when compared with warfarin, apixaban presented better preventive effects of ADVT on postoperative patients (OR 0.23, 95% CI 0.10–0.52). Warfarin showed worse preventive effects of ADVT on postoperative patients than ximelagatran and enoxaparin (OR 1.54, 95% CI 1.21–1.98; OR 2.35, 95% CI 1.16–4.76, respectively) (Table 1). In addition, when compared with enoxaparin, apixaban showed a relatively better effect in preventing CRNM bleeding (OR 0.66, 95% CI 0.45–0.97) (Table 1). Besides, there was no evident statistical difference when the direct paired comparison was conducted regarding the preventive effect of the 9 anticoagulant drugs on the symptomatic DVT, PE, and also major and minor bleeding in postoperative patients who received artificial joint replacement (Appendix Table 2). The evidence network diagram of the 9 anticoagulant drugs is shown in Fig. 3 and Appendix Fig. 2.

**Table 1:**
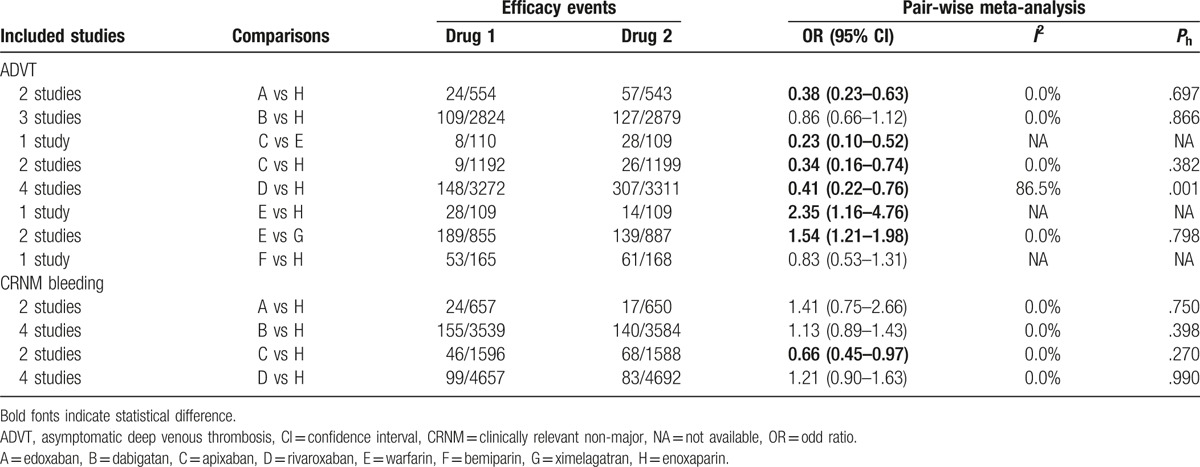
Pair-wise meta-analysis of ADVT and CRNM bleeding.

### 3.3 Major results of network meta-analysis

**Figure 4:**
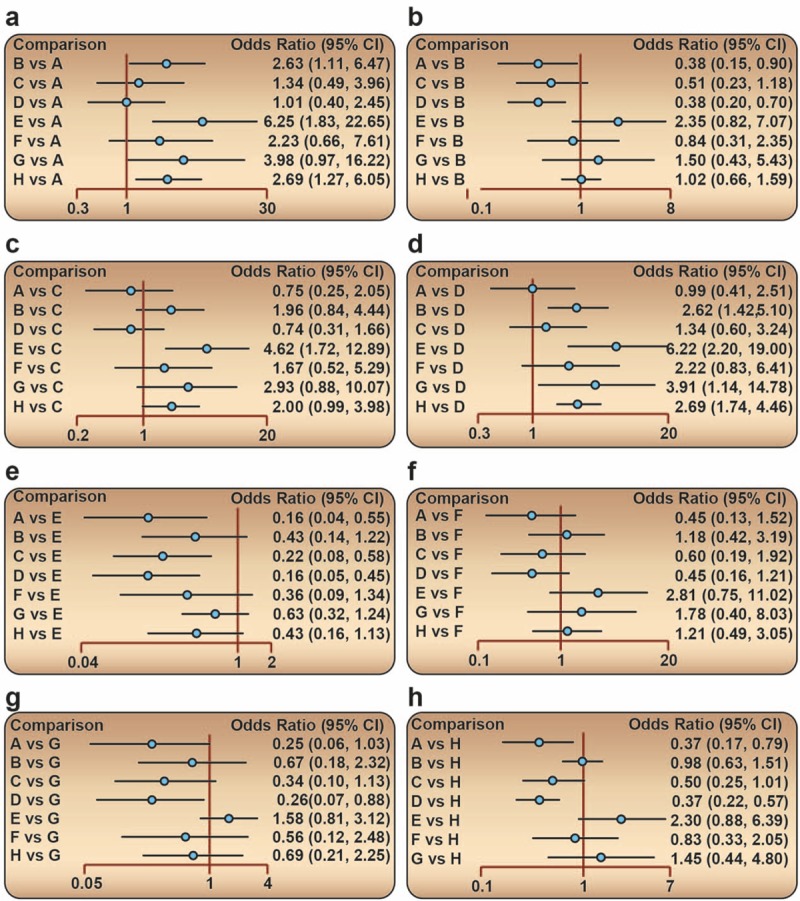
Forest plots for the relationship of the effect of different anticoagulant drugs on the prevention of postoperative ADVT in patients after arthroplasty. (A) Edoxaban; (B) dabigatan; (C) apixaban; (D) rivaroxaban; (E) warfarin; (F) bemiparin; (G) ximelagatran; (H) enoxaparin. ADVT = asymptomatic deep venous thrombosis.

**Figure 5:**
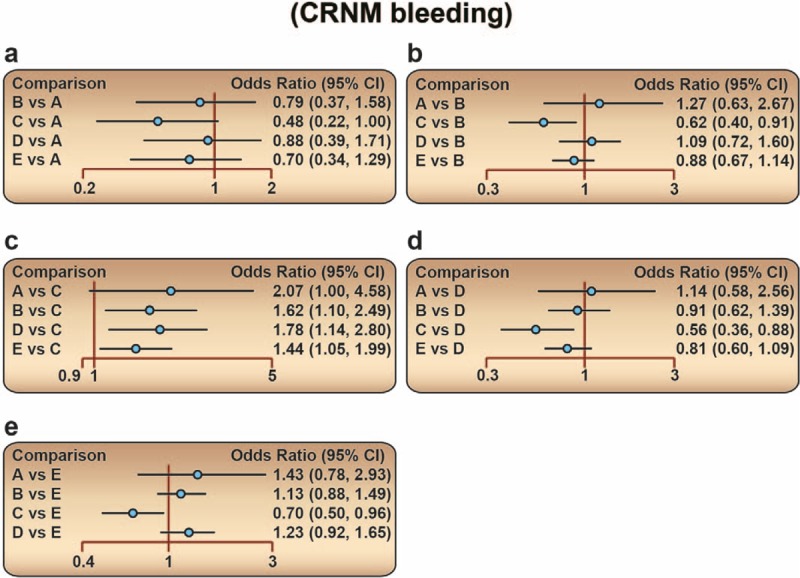
Forest plots for the relationship of the effect of different anticoagulant drugs on the prevention of postoperative CRNM bleeding in patients after arthroplasty. (A) Edoxaban; (B) dabigatan; (C) apixaban; (D) rivaroxaban; (E) enoxaparin. CRNM = clinically relevant non-major.

Our network meta-analysis indicated that edoxaban, apixaban, and rivaroxaban showed significantly lower incidence of ADVT of postoperative patients when compared with warfarin (OR 0.16, 95% CI 0.04–0.55; OR 0.22, 95% CI 0.08–0.58; OR 0.16, 95% CI 0.05–0.45, respectively), which indicated that the preventive effects of edoxaban, apixaban, and rivaroxaban were accordingly better. Further, when compared with enoxaparin, edoxaban and rivaroxaban also presented better preventive effects (OR 0.37, 95% CI 0.17–0.79; OR 0.37, 95% CI 0.22–0.57, respectively). Compared with dabigatan, similar results of better effect were also observed in patients treated with edoxaban and rivaroxaban (OR 0.38, 95% CI 0.15–0.90; OR 0.38, 95% CI 0.20–0.70, respectively). Besides, rivaroxaban also exhibited better effect when compared with ximelagatran (OR 0.26, 95% CI 0.07–0.88) (Table 2 and Fig. 4). With respect to CRNM bleeding, edoxaban, dabigatan, rivaroxaban, and enoxaparin treatment showed worse preventive effect than apixaban (OR 2.07, 95% CI 1.00–4.58; OR 1.62, 95% CI 1.10–2.49; OR 1.78, 95% CI 1.14–2.80; OR 1.44, 95% CI 1.05–1.99, respectively) (Table 3 and Fig. 5). However, there was no statistical difference regarding the preventive effect of the 9 drugs on the symptomatic DVT, PE, and also major and minor bleeding in postoperative patients who received artificial joint replacement (Appendix Table 3).

**Table 2:**
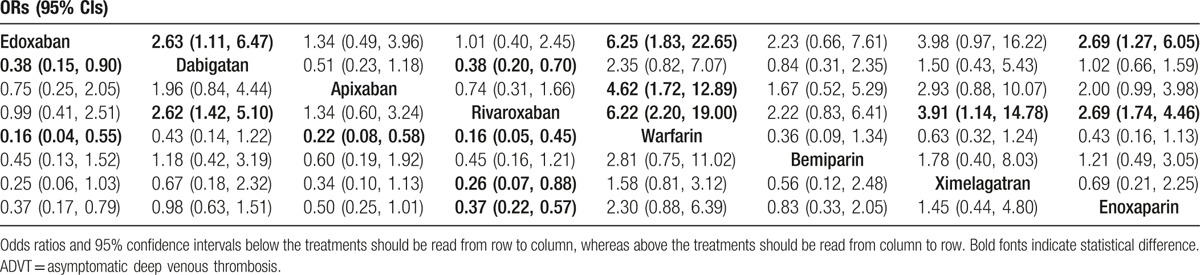
Odds ratios (ORs) and 95% confidence intervals (CIs) of 8 drugs for the prevention of ADVT.

**Table 3:**
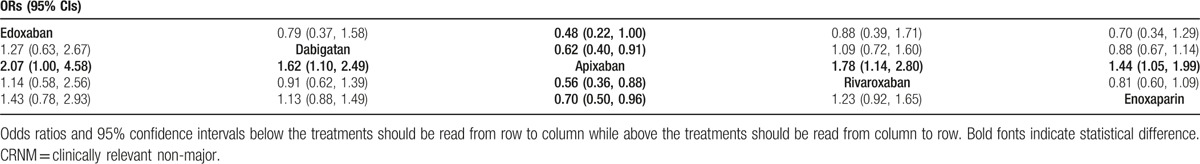
Odds ratios (ORs) and 95% confidence intervals (CIs) of 5 drugs for the prevention of CRNM bleeding.

### 3.4 Ranking of interventions

As shown in Table 4, the treatment-relative ranking of estimated probabilities concerning SUCRA values revealed that, in terms of edoxaban, it ranked highest under the SDVT and CRNM bleeding (SDVT: 87.8%; CRNM bleeding: 79.8%), whereas dabigatan was the lowest (PE: 74.2%; minor bleeding: 31.7%). Apixaban was the lowest under the SUCRA values of CRNM bleeding (1.3%). With respect to rivaroxaban, it ranked highest under the major bleeding, whereas it was the lowest under the ADVT and PE (major bleeding: 86.8%; ADVT: 11.3%; PE: 32.3%). Warfarin was the highest under the SUCRA values of ADVT and the lowest under the major bleeding (ADVT: 96.4%; major bleeding: 22.3%). As for heparin, it was the highest under the minor bleeding (93.8%), whereas bemiparin ranked the lowest under the SDVT (26.0%). In conclusion, in terms of SDVT and PE, rivaroxaban and bemiparin had better preventive effects, whereas apixaban and warfarin had better preventive effects on bleeding.

**Table 4:**
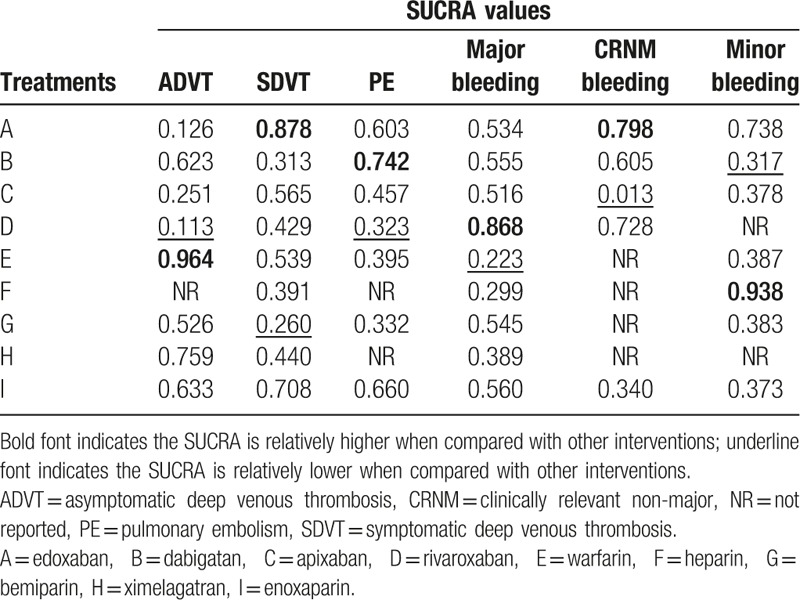
SUCRA values of 9 treatment modalities under 6 endpoint outcomes.

### 3.5 Meta-regression analyses

Postoperative patients were assigned into Asians, Caucasians, and mixed population for meta-regression analyses. Then, interventions were re-ordered and their SUCRA values were calculated, which is shown in Table 4 and Appendix Table 4. Concerning CRNM bleeding, the SUCRA values of edoxaban, dabigatan, apixaban, rivaroxaban, and enoxaparin changed 79.8%, 60.5%, 1.3%, 72.8%, and 34.0% into 76.3%, 70.4%, 24.6%, 78.8%, and 49.9%, respectively. The results demonstrated that the ranking of interventions with regard to the SDVT, ADVT, PE, major bleeding, and minor bleeding did not differ, which demonstrated that there were no significant differences in patients of different races.

## 4 Discussion

In this study, both the pair-wise meta-analysis and network meta-analysis revealed that the preventive efficacy of ADVT of warfarin and enoxaparin in patients after arthroplasty was better, whereas that of apixaban in patients after arthroplasty was relatively poor. Similarly, Shao et al^[41]^ analyzed in their study that when compared with warfarin, apixaban, edoxaban, and rivaroxaban had poorer preventive efficacy on ADVT of lower extremities after arthroplasty, which was similar to the corresponding results of the current study in this issue. Meanwhile, the preventive efficacy of rivaroxaban and edoxaban was also suggested to be poorer, when compared with enoxaparin.^[41]^ Generally, warfarin can minimize the tendency for thrombosis or is used as secondary prophylaxis which can prevent further episodes in individuals who have formed thrombus. It therefore might be reasonable to choose warfarin when an oral anticoagulant is needed.^[42]^ As for enoxaparin, together with antithrombin (a circulating anticoagulant), it could form a complex, irreversibly prohibiting clotting factor become active, which might therefore have bioavailability and possess predictable absorption characteristics. On the contrary, apixaban in ADVT prophylaxis may cause PE in hip or knee replacement surgery patients; hence it might be responsible for the poor efficacy in those patients after arthroplasty.^[43]^

As is evident from the results of SUCRA, warfarin had better preventive efficacy on ADVT. Although warfarin and enoxaparin were both better than other drugs, edoxaban may have adverse effects, such as unusual bleeding or bruising. With respect to minor bleeding, heparin, the widely used injectable blood thinner, was relatively better, because it produced anticoagulative action that prevented the extension of the existing clots and the formation of clots within the blood. In patients with high risk of thrombosis, when the anticoagulant is withdrawn, heparin is suggested.^[42]^ The data indicated that, after arthroplasty, the patients with complications exhibited differed responses to different drugs.

There were several limitations of this research that should be cautiously taken into consideration. Firstly, because the significant differences of the sample sizes on which all these 9 drugs were tested were relatively large and the number of them by direct paired comparison in this study were not the same, to some degree, it might affect the results. Secondly, the data analyses were rich in content, but because of the prevention effect of each drug in different complications, it failed to undergo cluster analysis. Even though, there were advantages in this study that should be pointed out, namely, all the subjects included in the study were clinical patients after artificial joint replacement, so the study had an important clinical significance on postoperative orthopedic surgery, whether it will cause different types of complications or not.

## 5 Conclusions

In summary, the current network meta-analysis provides evidence that among these 9 kinds of anticoagulant drugs, warfarin and enoxaparin have a relatively good prevention effect on postoperative ADVT for patients with arthroplasty, and apixaban has a relatively bad effect on preventing arthroplasty patients from suffering from CRNM bleeding. These findings have provided reference for developing drugs and management of arthroplasty-related complications in the future. However, some limitations still remain in our study. First, arthroplasty surgery is indicated in diseases from diverse etiologies, which may cause variations in coagulative state and response to different anticoagulants. But detailed data about the disease history or former situation are lacking on those patients in enrolled studies. Therefore, we cannot perform subgroup analysis and heterogeneity analysis based on the etiologies. Although high-quality studies were included, this still may influence our results. Second, due to the lack of detailed information of each enrolled patient, such as specific disease situation and prognosis data, we cannot give more clinical suggestions, except our conclusions, in this study. Therefore, our conclusion needs to be further confirmed based on higher-quality RCTs with more detailed and complete information because of the above limitations.

## Acknowledgment

The authors wish to express their gratitude to reviewers for their critical comments.

## Supplementary Material

Supplemental Digital Content

## Supplementary Material

Supplemental Digital Content
