# Supplementary material for: Effects of different anticoagulant drugs on the prevention of complications in patients after arthroplasty: A network meta-analysis
Source: Medicine (Baltimore). 2017 Oct 27;96(40):e8059. doi: 10.1097/MD.0000000000008059 (PMC5737997; doi:10.1097/MD.0000000000008059)
Supplement: Supplemental Digital Content [file medi-96-e8059-s001.doc]

**Appendix Table 1. The baseline characteristics of included studies.**

| **First author** | **Year** | **Ethnicity** | **Treatments** | | | **Total** | **Sample size** | | | **Gender (M/F)** | | | **Age (years)** | | |
| --- | --- | --- | --- | --- | --- | --- | --- | --- | --- | --- | --- | --- | --- | --- | --- |
|  |  |  | **D1** | **D2** | **D3** |  | **D1** | **D2** | **D3** | **D1** | **D2** | **D3** | **D1** | **D2** | **D3** |
| Fuji T | 2015 | Asians | A | I | - | 503 | 255 | 248 | - | 35/220 | 36/212 | - | 62.8 ± 9.61 | 62.8 ± 9.72 | - |
| Mirdamadi A | 2014 | Asians | B | I | - | 90 | 45 | 45 | - | 17/28 | 15/30 | - | 72.1±9.3 | 68.3 ± 10.1 | - |
| Fuji T | 2014 | Asians | A | I | - | 594 | 299 | 295 | - | 54/245 | 66/229 | - | 72.6±7.5 | 72.1±7.8 | - |
| Eriksson BI | 2011 | mixed population | B | I | - | 2013 | 1010 | 1003 | - | 469/541 | 502/501 | - | 62.0±12.0 | 62.0±11.0 | - |
| Lassen MR | 2010 | mixed population | C | I | - | 3057 | 1528 | 1529 | - | 439/1089 | 402/1127 | - | 67 (59-73) | 67 (60-73) | - |
| Friedman RJ | 2010 | mixed population | B | I | - | 4672 | 2076 | 2596 | - | 706/1370 | 1091/1595 | - | 68.0±9.0 | 66.0±10.0 | - |
| Turpie AG | 2009 | mixed population | D | I | - | 3034 | 1526 | 1508 | - | 519/1007 | 541/967 | - | 64.4±9.7 | 64.7±9.7 | - |
| Lassen MR | 2009 | mixed population | C | I | - | 3195 | 1599 | 1596 | - | 602/997 | 610/986 | - | 65.9 (26-93) | 65.7 (33-89) | - |
| Ginsberg JS | 2009 | Caucasians | B | I | - | 1725 | 857 | 868 | - | 371/486 | 350/507 | - | 66.2 ± 9.5 | 66.3 ± 9.6 | - |
| Lassen MR | 2008 | mixed population | D | I | - | 2459 | 1220 | 1239 | - | 363/857 | 418/821 | - | 67.6 (28-91) | 67.6 (30-90) | - |
| Kakkar AK | 2008 | mixed population | D | I | - | 2457 | 1228 | 1229 | - | 561/667 | 578/651 | - | 61.4 (18-93) | 61.6 (19-93) | - |
| Eriksson BI | 2008 | mixed population | D | I | - | 4433 | 2209 | 2224 | - | 989/1220 | 982/1242 | - | 63.1 (18-91) | 63.3 (18-93) | - |
| Lassen MR | 2007 | mixed population | C | E | I | 461 | 156 | 152 | 153 | 55/101 | 58/94 | 60/93 | 65.8 (35-90) | 66.5 (36-88) | 66.8 (43-85) |
| Eriksson BI | 2007 | mixed population | B | I | - | 1373 | 679 | 694 | - | - | - | - | 67.0 ± 9.0 | 68.0 ± 9.0 | - |
| Senaran H | 2006 | Caucasians | F | I | - | 100 | 50 | 50 | - | 17/33 | 12/38 | - | 52.4±11.2 | 55.2±8.4 | - |
| Navarro-Quilis A | 2003 | Caucasians | G | I | - | 380 | 189 | 191 | - | 44/145 | 32/159 | - | 70.5±6.6 | 68.9±6.4 | - |
| Francis CW | 2003 | mixed population | E | H | - | 1528 | 759 | 769 | - | 292/465 | 277/492 | - | 67.8±9.6 | 68.5±9.5 | - |
| Colwell CW | 2003 | mixed population | H | I | - | 1557 | 782 | 775 | - | 372/410 | 377/398 | - | 64.5±12.8 | 64.0±13.1 | - |
| Francis CW | 2002 | mixed population | E | H | - | 675 | 330 | 345 | - | 118/212 | 127/218 | - | 67.7±10.4 | 67.8±10.1 | - |

D=drug; M=male; F=female; A=Edoxaban; B=Dabigatan; C=Apixaban; D=Rivaroxaban; E=Warfarin; F=Heparin; G=Bemiparin; H=Ximelagatran; I=Enoxaparin.

**Appendix Table 2. Pairwise meta-analysis of SDVT, PE, major bleeding and minor bleeding.**

| **Included studies** | **Comparisons** | **Efficacy events** | |  | **Pairwise meta-analysis** | | |
| --- | --- | --- | --- | --- | --- | --- | --- |
|  |  | **Drug1** | **Drug2** |  | **OR (95%CI)** | ***I*^2^** | ***P_h_*** |
| **SDVT** | | | | | | | |
| 2 studies | A vs. I | 5/554 | 2/543 |  | 2.32(0.41-12.97) | 0.0% | 0.435 |
| 3 studies | B vs. I | 8/1046 | 5/1037 |  | 0.42(0.07-2.36) | 0.0% | 0.441 |
| 1 study | C vs. E | 1/110 | 1/109 |  | 0.99(0.06-16.04) | NA | NA |
| 2 studies | C vs. I | 3/1528 | 7/1529 |  | 0.43(0.11-1.66) | 0.0% | 0.421 |
| 4 studies | D vs. I | 14/3997 | 28/3988 |  | 0.56(0.18-1.70) | 0.0% | 0.082 |
| 2 studies | E vs. H | 23/1017 | 19/1043 |  | 1.26(0.68-2.34) | 0.0% | 0.493 |
| 1 study | E vs. I | 1/109 | 1/109 |  | 1.00(0.06-16.19) | NA | NA |
| 1 study | F vs. I | 1/50 | 2/50 |  | 0.49(0.04-5.58) | NA | NA |
| 1 study | G vs. I | 2/165 | 7/168 |  | 0.28(0.06-1.38) | NA | NA |
| **PE** | | | | | | | |
| 2 studies | A vs. I | 2/554 | 2/543 |  | 0.98(0.14-6.98) | 0.0% | 0.994 |
| 5 studies | B vs. I | 17/2890 | 13/2964 |  | 1.34(0.65-2.77) | 0.0% | 0.840 |
| 1 study | C vs. E | 1/110 | 1/109 |  | 0.99(0.06-16.04) | NA | NA |
| 2 studies | C vs. I | 2/1562 | 5/1554 |  | 0.20(0.02-1.70) | 16.9% | 0.307 |
| 2 studies | D vs. I | 6/2727 | 12/2725 |  | 0.51(0.19-1.39) | 0.0% | 0.478 |
| 1 study | E vs. I | 1/109 | 2/109 |  | 0.50(0.04-5.54) | NA | NA |
| 1 study | F vs. I | 1/165 | 2/168 |  | 0.51(0.05-5.64) | NA | NA |
| **Major bleeding** | | | | | | | |
| 2 studies | A vs. I | 6/657 | 7/650 |  | 1.01(0.09-11.62) | 11.4% | 0.072 |
| 5 studies | B vs. I | 55/3737 | 50/3764 |  | 1.11(0.75-1.63) | 0.0% | 0.609 |
| 1 study | C vs. E | 5/151 | 1/151 |  | 5.14(0.59-44.50) | NA | NA |
| 1 study | C vs. I | 5/151 | 1/149 |  | 5.07(0.59-43.91) | NA | NA |
| 3 studies | D vs. I | 18/3974 | 11/3976 |  | 1.61(0.75-3.47) | 0.0% | 0.624 |
| 2 studies | E vs. H | 6/1089 | 8/1114 |  | 0.77(0.26-2.23) | 0.0% | 0.725 |
| 1 study | E vs. I | 1/151 | 1/149 |  | 0.99(0.06-15.92) | NA | NA |
| 1 study | F vs. I | 1/50 | 2/50 |  | 0.49(0.04-5.58) | NA | NA |
| 1 study | G vs. I | 3/189 | 3/191 |  | 1.01(0.20-5.07) | NA | NA |
| 1 study | H vs. I | 7/906 | 8/910 |  | 0.88(0.32-2.43) | NA | NA |
| **Minor bleeding** | | | | | | | |
| 1 study | A vs. I | 57/303 | 39/301 |  | 1.56(1.00-2.42) | NA | NA |
| 3 studies | B vs. I | 245/3737 | 255/3764 |  | 0.97(0.81-1.16) | 0.0% | 0.591 |
| 1 study | C vs. E | 10/151 | 8/151 |  | 1.27(0.49-3.31) | NA | NA |
| 2 studies | C vs. I | 49/1747 | 46/1737 |  | 1.06(0.70-1.59) | 0.0% | 0.335 |
| 1 study | E vs. I | 8/151 | 6/149 |  | 1.33(0.45-3.94) | NA | NA |
| 1 study | F vs. I | 4/50 | 1/50 |  | 4.26(0.46-39.54) | NA | NA |
| 1 study | G vs. I | 4/189 | 4/191 |  | 1.01(0.25-4.10) | NA | NA |

Notes: OR=odd ratios; CI=confidence intervals; NA=not available; SDVT=symptomatic deep venous thrombosis; PE= pulmonary embolism. A=Edoxaban; B=Dabigatan; C=Apixaban; D=Rivaroxaban; E=Warfarin; F=Heparin; G=Bemiparin; H=Ximelagatran; I=Enoxaparin.

| **Appendix Table 3**. Odds ratios and 95% confidence intervals of nine drugs for the prevention of SDVT, PE, Major bleeding and Minor bleeding. | | | | | | | | |
| --- | --- | --- | --- | --- | --- | --- | --- | --- |
| **Odds ratio (95%CI)** | | | | | | | | |
| SDVT | | | | | | | | |
| Edoxaban | 0.15 (0.01, 1.34) | 0.27 (0.02, 2.93) | 0.20 (0.02, 1.68) | 0.25 (0.00, 8.61) | 0.14 (0.00, 5.43) | 0.09 (0.00, 1.54) | 0.19 (0.00, 8.37) | 0.38 (0.04, 2.61) |
| 6.77 (0.75, 87.84) | Dabigatan | 1.83 (0.34, 11.45) | 1.31 (0.35, 6.23) | 1.76 (0.03, 40.22) | 0.97 (0.03, 22.87) | 0.65 (0.05, 6.99) | 1.37 (0.02, 39.16) | 2.51 (0.87, 8.83) |
| 3.65 (0.34, 48.66) | 0.55 (0.09, 2.92) | Apixaban | 0.72 (0.15, 3.66) | 0.91 (0.02, 16.07) | 0.52 (0.01, 12.39) | 0.35 (0.02, 4.10) | 0.71 (0.01, 15.13) | 1.36 (0.35, 5.18) |
| 5.06 (0.60, 58.87) | 0.76 (0.16, 2.83) | 1.39 (0.27, 6.88) | Rivaroxaban | 1.34 (0.03, 26.67) | 0.74 (0.02, 15.61) | 0.50 (0.04, 4.64) | 1.04 (0.02, 25.31) | 1.92 (0.77, 4.55) |
| 4.03 (0.12, 353.84) | 0.57 (0.02, 29.16) | 1.10 (0.06, 45.31) | 0.75 (0.04, 39.78) | Warfarin | 0.59 (0.00, 87.39) | 0.37 (0.01, 30.63) | 0.78 (0.25, 2.46) | 1.41 (0.08, 60.91) |
| 6.92 (0.18, 478.25) | 1.03 (0.04, 39.81) | 1.92 (0.08, 94.01) | 1.36 (0.06, 58.00) | 1.70 (0.01, 208.36) | Heparin | 0.69 (0.02, 38.89) | 1.33 (0.01, 179.52) | 2.54 (0.13, 98.69) |
| 10.62 (0.65, 235.28) | 1.54 (0.14, 18.57) | 2.83 (0.24, 42.24) | 2.00 (0.22, 24.60) | 2.67 (0.03, 93.83) | 1.45 (0.03, 60.52) | Bemiparin | 2.14 (0.02, 93.51) | 3.81 (0.49, 39.75) |
| 5.23 (0.12, 474.00) | 0.73 (0.03, 43.13) | 1.41 (0.07, 73.07) | 0.96 (0.04, 60.44) | 1.27 (0.41, 3.96) | 0.75 (0.01, 104.61) | 0.47 (0.01, 40.55) | Ximelagatran | 1.87 (0.08, 96.50) |
| 2.64 (0.38, 24.95) | 0.40 (0.11, 1.15) | 0.73 (0.19, 2.84) | 0.52 (0.22, 1.29) | 0.71 (0.02, 12.38) | 0.39 (0.01, 7.47) | 0.26 (0.03, 2.06) | 0.54 (0.01, 11.94) | Enoxaparin |
| PE | | | | | | | | |
| Edoxaban | 1.19 (0.09, 13.83) | 0.64 (0.04, 8.99) | 0.49 (0.03, 6.21) | 0.49 (0.01, 14.58) | 0.35 (0.01, 12.86) | 1.04 (0.09, 10.35) |  | |
| 0.84 (0.07, 11.03) | Dabigatan | 0.58 (0.10, 2.88) | 0.41 (0.08, 1.85) | 0.42 (0.01, 5.27) | 0.30 (0.01, 5.86) | 0.85 (0.36, 2.17) |
| 1.56 (0.11, 23.79) | 1.72 (0.35, 9.74) | Apixaban | 0.72 (0.11, 4.15) | 0.76 (0.03, 9.56) | 0.53 (0.01, 11.26) | 1.51 (0.42, 6.25) |
| 2.02 (0.16, 34.02) | 2.44 (0.54, 12.60) | 1.38 (0.24, 9.13) | Rivaroxaban | 1.00 (0.03, 17.72) | 0.83 (0.01, 17.20) | 2.15 (0.66, 8.84) |
| 2.04 (0.07, 97.96) | 2.41 (0.19, 79.95) | 1.31 (0.10, 34.58) | 1.00 (0.06, 35.30) | Warfarin | 0.79 (0.01, 47.00) | 2.02 (0.20, 68.71) |
| 2.83 (0.08, 180.76) | 3.35 (0.17, 140.01) | 1.87 (0.09, 100.59) | 1.20 (0.06, 74.31) | 1.27 (0.02, 109.15) | Bemiparin | 2.61 (0.18, 108.04) |
| 0.96 (0.10, 10.84) | 1.18 (0.46, 2.77) | 0.66 (0.16, 2.40) | 0.46 (0.11, 1.51) | 0.50 (0.01, 5.01) | 0.38 (0.01, 5.68) | Enoxaparin |
| **Minor bleeding** | | | | | | | | |
| Edoxaban | 0.63 (0.32, 1.19) | 0.65 (0.31, 1.31) | 0.64 (0.20, 2.02) | 4.87 (0.44, 264.66) | 0.61 (0.11, 2.61) | 0.66 (0.35, 1.15) |  | |
| 1.58 (0.84, 3.15) | Dabigatan | 1.05 (0.67, 1.71) | 1.02 (0.37, 3.00) | 7.88 (0.79, 475.14) | 0.99 (0.18, 3.88) | 1.03 (0.77, 1.36) |
| 1.53 (0.76, 3.17) | 0.96 (0.59, 1.50) | Apixaban | 0.98 (0.36, 2.69) | 7.32 (0.72, 419.51) | 0.95 (0.17, 3.82) | 1.00 (0.66, 1.41) |
| 1.56 (0.50, 4.91) | 0.98 (0.33, 2.69) | 1.02 (0.37, 2.76) | Warfarin | 7.96 (0.66, 453.36) | 0.95 (0.11, 5.27) | 1.01 (0.36, 2.75) |
| 0.21 (0.00, 2.29) | 0.13 (0.00, 1.27) | 0.14 (0.00, 1.39) | 0.13 (0.00, 1.51) | Heparin | 0.14 (0.00, 1.68) | 0.13 (0.00, 1.28) |
| 1.64 (0.38, 9.24) | 1.01 (0.26, 5.58) | 1.05 (0.26, 5.85) | 1.06 (0.19, 8.73) | 7.02 (0.60, 465.90) | Bemiparin | 1.04 (0.28, 5.49) |
| 1.52 (0.87, 2.85) | 0.97 (0.73, 1.29) | 1.00 (0.71, 1.52) | 0.99 (0.36, 2.77) | 7.49 (0.78, 441.07) | 0.96 (0.18, 3.62) | Enoxaparin |
| **Major bleeding** | | | | | | | | |
| Edoxaban | 1.05 (0.21, 5.36) | 0.92 (0.18, 7.28) | 2.02 (0.35, 11.78) | 0.46 (0.06, 3.90) | 0.39 (0.01, 9.50) | 1.09 (0.09, 13.47) | 0.71 (0.11, 5.47) | 1.06 (0.25, 4.41) |
| 0.95 (0.19, 4.69) | Dabigatan | 0.91 (0.27, 4.54) | 1.97 (0.59, 6.25) | 0.45 (0.08, 2.28) | 0.37 (0.01, 7.41) | 0.99 (0.11, 9.47) | 0.71 (0.15, 3.42) | 1.01 (0.50, 2.05) |
| 1.08 (0.14, 5.60) | 1.09 (0.22, 3.77) | Apixaban | 2.09 (0.39, 8.36) | 0.51 (0.06, 2.33) | 0.38 (0.01, 8.87) | 1.10 (0.09, 11.03) | 0.77 (0.12, 3.65) | 1.11 (0.28, 3.10) |
| 0.49 (0.08, 2.85) | 0.51 (0.16, 1.69) | 0.48 (0.12, 2.56) | Rivaroxaban | 0.23 (0.03, 1.36) | 0.19 (0.01, 3.99) | 0.51 (0.05, 5.34) | 0.36 (0.07, 2.00) | 0.52 (0.20, 1.35) |
| 2.16 (0.26, 16.81) | 2.23 (0.44, 13.33) | 1.97 (0.43, 15.60) | 4.28 (0.73, 29.51) | Warfarin | 0.84 (0.02, 23.39) | 2.25 (0.19, 33.31) | 1.54 (0.46, 6.08) | 2.26 (0.50, 11.50) |
| 2.54 (0.11, 112.10) | 2.69 (0.13, 89.76) | 2.60 (0.11, 103.57) | 5.29 (0.25, 189.34) | 1.18 (0.04, 51.66) | Heparin | 2.97 (0.08, 150.59) | 1.95 (0.08, 77.83) | 2.73 (0.15, 86.16) |
| 0.92 (0.07, 11.37) | 1.01 (0.11, 8.76) | 0.91 (0.09, 10.72) | 1.96 (0.19, 18.56) | 0.44 (0.03, 5.15) | 0.34 (0.01, 13.30) | Bemiparin | 0.71 (0.06, 8.14) | 1.01 (0.12, 7.87) |
| 1.41 (0.18, 9.36) | 1.41 (0.29, 6.59) | 1.29 (0.27, 8.58) | 2.77 (0.50, 14.66) | 0.65 (0.16, 2.16) | 0.51 (0.01, 12.33) | 1.41 (0.12, 17.57) | Ximelagatran | 1.42 (0.36, 5.57) |
| 0.94 (0.23, 3.94) | 0.99 (0.49, 1.98) | 0.90 (0.32, 3.58) | 1.94 (0.74, 5.07) | 0.44 (0.09, 1.99) | 0.37 (0.01, 6.73) | 0.99 (0.13, 8.56) | 0.70 (0.18, 2.75) | Enoxaparin |
| Notes: SDVT, symptomatic deep venous thrombosis; PE, pulmonary embolish. | | | | | | | | |

**Appendix Table 4. SUCRA values of nine drugs under six endpoint outcomes after meta-**regression analysis.

| **Treatments** | **SUCRA values** | | | | | |
| --- | --- | --- | --- | --- | --- | --- |
|  | **ADVT** | **SDVT** | **PE** | **Major bleeding** | **CRNM bleeding** | **Minor bleeding** |
| **A** | 0.443 | **0.784** | 0.600 | 0.584 | 0.763 | 0.615 |
| **B** | 0.626 | 0.410 | **0.771** | 0.603 | 0.704 | 0.451 |
| **C** | 0.324 | 0.619 | 0.532 | 0.554 | 0.246 | 0.513 |
| **D** | 0.220 | 0.502 | 0.443 | **0.854** | **0.788** | NR |
| **E** | **0.945** | 0.605 | 0.462 | 0.329 | NR | 0.493 |
| **F** | NR | 0.471 | NR | 0.376 | NR | **0.919** |
| **G** | 0.545 | 0.335 | 0.473 | 0.605 | NR | 0.504 |
| **H** | 0.754 | 0.524 | NR | 0.493 | NR | NR |
| **I** | 0.643 | 0.749 | 0.720 | 0.603 | 0.499 | 0.504 |

Notes: ADVT=asymptomatic deep venous thrombosis; SDVT= symptomatic deep venous thrombosis; PE= pulmonary embolism; CRNM= clinically relevant non-major; NR=not report; A=Edoxaban; B=Dabigatan; C=Apixaban; D=Rivaroxaban; E=Warfarin; F=Heparin; G=Bemiparin; H=Ximelagatran; I=Enoxaparin. Bold font, the SUCRA is relatively higher when compared with other interventions; Underline font, the SUCRA is relatively lower when compared with other interventions.
