# Supplementary material for: Effects of different anticoagulant drugs on the prevention of complications in patients after arthroplasty: A network meta-analysis
Source: Medicine (Baltimore). 2017 Oct 27;96(40):e8059. doi: 10.1097/MD.0000000000008059 (PMC5737997; doi:10.1097/MD.0000000000008059)
Supplement: Supplemental Digital Content [file medi-96-e8059-s002.doc]

**Search Strategy**

#1 Anticoagulants[mesh] OR Anticoagulation Agents[tiab] OR Agents, Anticoagulation[tiab] OR Anticoagulant Agents[tiab] OR Anticoagulant Drugs[tiab] OR Drugs, Anticoagulant[tiab] OR Anticoagulant[tiab] OR Indirect Thrombin Inhibitors[tiab] OR Inhibitors, Indirect Thrombin[tiab] OR apixaban[tiab] OR enoxaparin[tiab] OR rivaroxaban[tiab] OR Savaysa[tiab] OR warfarin[tiab] OR edoxaban[tiab] OR edoxaban[tiab] OR bemiparin[tiab] OR dicoumarin[tiab] OR dabigatan[tiab] OR heparin[tiab] OR dicumarol[tiab] OR Dipyridamole[tiab] OR ximelagatran[tiab] OR acenocoumarol[tiab] OR argatroban[tiab] OR fondaparinux[tiab] OR idraparinux[tiab] OR enoxaparin[tiab]

#2 Arthroplasty[mesh] OR Arthroplasties[tiab] OR Arthroplasty, Replacement, Knee[mesh] OR Arthroplasty, Knee Replacement[tiab] OR Knee Replacement Arthroplasties[tiab] OR Knee Replacement Arthroplasty[tiab] OR Total Knee Arthroplasty[tiab] OR Total Knee Replacement[tiab] OR Knee Replacement, Total[tiab] OR Knee Arthroplasty[tiab] OR Arthroplasties, Knee Replacement[tiab] OR Replacement Arthroplasty, Knee[tiab] OR Unicompartmental Knee Arthroplasty[tiab] OR Unicondylar Knee Arthroplasty[tiab] OR Partial Knee Arthroplasty[tiab] OR Arthroplasty, Partial Knee[tiab] OR Unicondylar Knee Replacement[tiab] OR Partial Knee Replacement[tiab] OR Unicompartmental Knee Replacement[tiab] OR Arthroplasty, Replacement, Hip[mesh] OR Arthroplasties, Replacement, Hip[tiab] OR Arthroplasty, Hip Replacement[tiab] OR Hip Prosthesis Implantation[tiab] OR Implantation, Hip Prosthesis[tiab] OR Prosthesis Implantation, Hip[tiab] OR Hip Replacement Arthroplasty[tiab] OR Replacement Arthroplasty, Hip[tiab] OR Arthroplasties, Hip Replacement[tiab] OR Hip Replacement Arthroplasties[tiab] OR Hip Replacement, Total[tiab] OR Replacements, Total Hip[tiab] OR Total Hip Replacement[tiab]

#3 "Venous Thrombosis"[mh] OR lower extremity deep venous thrombosis[tiab] OR lower extremity DVT[tiab] OR deep venous thrombosis[tiab] OR deep vein thrombosis[tiab] OR deep vein thrombus[tiab] OR DVT[tiab]

#4 "randomized controlled trial"[pt] OR "controlled clinical trial"[pt] OR "randomized controlled trials as topic"[Mesh] OR "clinical trials as topic"[mh] OR "controlled clinical trials as topic"[mh] OR placebos[mh] OR "random allocation"[mh] OR “double-blind method"[mh] OR randomized[tiab] OR placebo[tiab] OR randomization[tiab] OR randomly allocated[tiab] OR ((double[tw] OR treble[tw] OR triple[tw]) AND (mask* [tw] OR blind* [tw]))

#5 “meta-analysis"[pt] OR “meta-analysis as topic"[mh] OR meta-analysis[tiab] OR network meta-analysis[tiab] OR mixed treatment comparison*[tiab] OR multiple treatment comparison*[tiab] OR multiple treatment meta-analysis[tiab]


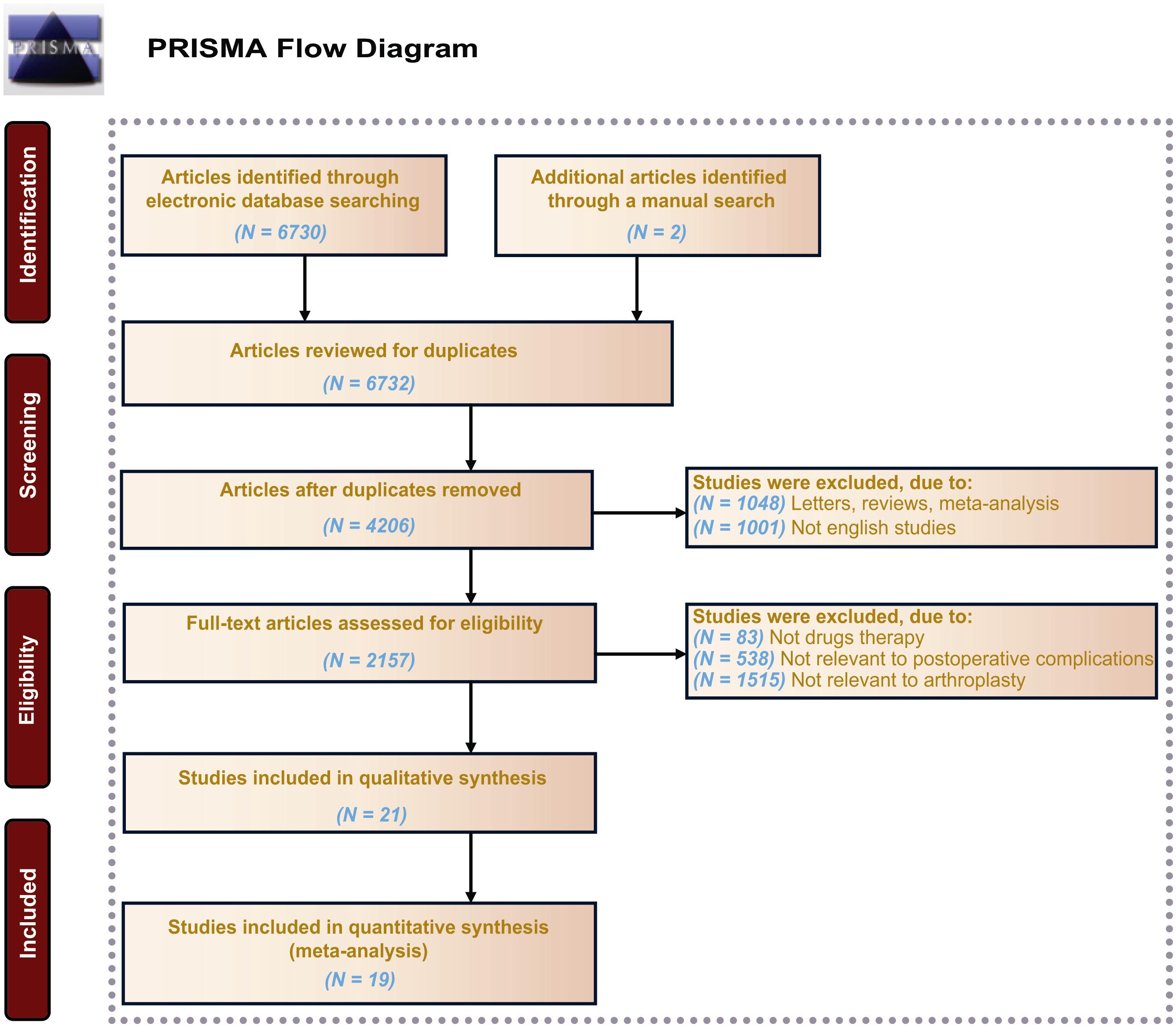


**Appendix Figure 1.** Flow chart of literature screening process. Combined with electronic literature identification and predetermined inclusion criteria screening, a total of nineteen studies were incorporated into our network meta-analysis.

**
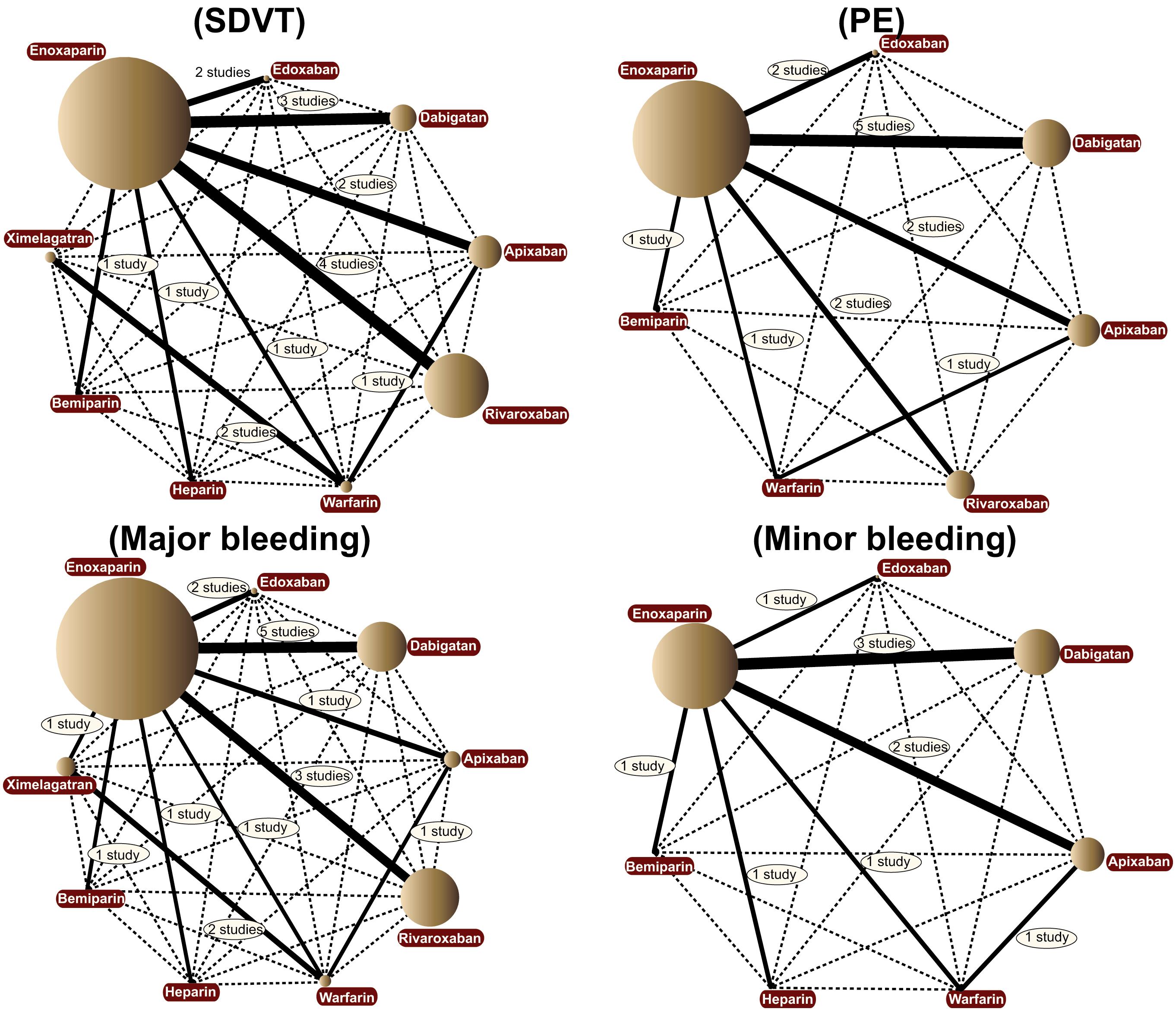
**

**Appendix Figure 2.** The evidence network of all enrolled studies about the preventive effect of the nine anticoagulant drugs (edoxaban, dabigatan, apixaban, rivaroxaban, warfarin, heparin, bemiparin, ximelagatran and enoxaparin) on the symptomatic deep venous thrombosis, pulmonary embolism, as well as major and minor bleeding in post-operative patients received arthroplasty in this network meta-analysis.

Note: SDVT = symptomatic deep venous thrombosis; PE = pulmonary embolism
